# Effect of continuous chelation on the dentinal tubule penetration of a calcium silicate–based root canal sealer: a confocal laser microscopy study

**DOI:** 10.1186/s12903-023-02995-z

**Published:** 2023-06-09

**Authors:** Reham Hassan, Nehal Nabil Roshdy

**Affiliations:** 1 Egyptian Russian University, Badr city, Egypt; 2 Faculty of Dentistry, Minia University, Minia, Egypt; 3 Faculty of Dentistry, Cairo University, Giza, Egypt

**Keywords:** Bioceramic sealer, Confocal, Continuous chelation, EDTA, Dual Rinse HEDP, Sealer penetration

## Abstract

**Background:**

This study aimed to evaluate the effect of various irrigation protocols on the penetration depth of a calcium silicate–based sealer into dentinal tubules using confocal laser scanning microscopy (CLSM).

**Methods:**

Twenty single-rooted mandibular premolars were endodontically prepared and divided into the following two groups according to the irrigation protocol used (n = 10): Group I: NaOCl + EDTA and Group II: continuous chelation (NaOCl/Dual Rinse). Obturation was performed with the warm vertical compaction technique using TotalFill HiFlow bioceramic sealer mixed with a fluorophore dye. Samples were observed using CLSM at 10× to measure the percentage of sealer penetration and its maximum depth into the dentinal tubules. Data were analysed using one-way ANOVA followed by Tukey’s post-hoc test. The significance level was set at p < 0.05 within all tests.

**Results:**

Comparing the overall results of all sections tested, no statistically significant differences existed between the groups regarding the percentage of sealer penetration (p = 0.612) and maximum depth of penetration (p > 0.05).

**Conclusions:**

With both types of irrigation used, dentinal tubule penetration was higher in the coronal section than in the apical section. Continuous chelation using NaOCl/Dual Rinse HEDP performed better in the coronal segments, while irrigation using NaOCl + EDTA promoted a higher percentage of sealer penetration in the apical segment.

## Introduction

The goal of root canal treatment is to properly debride and shape the root canal space, which is then filled with an inert obturating material to block any routes of reinfection [1]. Mechanical instrumentation of the root canal is capable of producing a smear layer that covers the root canal walls [2]. An ideal endodontic irrigating solution should dissolve necrotic tissue, remove the smear layer, possess broad antimicrobial efficiency, and have low cytotoxicity. Until now, no single irrigant has been able to fulfil all four of these criteria [3].

Sodium hypochlorite (NaOCl) has a unique organic tissue-dissolving efficacy, is an effective antiseptic, and is minimally caustic at reduced concentrations [4]. These properties make NaOCl indispensable in root canal treatment. However, NaOCl solutions do not have any effect on the inorganic part of the smear layer, which blocks the dentinal tubules that incubate bacteria [3]. Additionally, the smear layer inhibits the adaptation of root canal sealers to intraradicular dentin walls [5].

It has been suggested that applying 17% ethylene diamine tetra acetic acid (EDTA) solution as a final rinse assists with the removal of the smear layer [6]. Since sodium hypochlorite and EDTA solutions cannot be mixed, the rinse involves two separate steps. Furthermore, mixing an oxidising agent (NaOCl) with a chelating agent, such as EDTA or citric acid, creates a chemical interaction and an exothermic reaction [7], which consume the available chlorine in NaOCl solutions and compromise its antimicrobial and tissue-dissolving properties [8].

Recently, Dual Rinse irrigating solution (Medcem, Weinfelden, Switzerland) was introduced to the market, which combines sodium hypochlorite with etidronic acid (1- hydroxyethane-1, 1-diphosphonic acid, HEDP) [9]. It is a powder in preweighed capsules that must be added to NaOCl rinsing solution just before use. HEDP is a chelating agent that exhibits stability and activity for 1 h when dissolved in sodium hypochlorite solution [10].

The ultimate benefit of using this novel irrigant is that both reagents are present throughout the cleaning and shaping procedures [11]. Over the past years, the concept of ‘continuous chelation’ [12] in the context of root canal therapy has gained wide acceptance in endodontic research as clinicians are currently looking into continuous chelation concept as a time-saving strategy. The proteolytic/antibacterial effects of NaOCl, which are mainly based on the free available chlorine, are maintained [9], while HEDP as a chelator (a calcium sequestering agent) prevents the build-up of a smear layer [13].

Among a wide spectrum of commercially available root canal sealers, TotalFill HiFlow bioceramic (BC) sealer (FKG Dentaire) is a premixed, ready-to‐use, injectable calcium phosphate silicate–based cement that can be used in warm filling techniques [14, 15]. Warm gutta-percha filling techniques demand the use of root canal sealers that can tolerate the heating process. Most of the available sealers suffers from property alterations when heated, which result in reduction of the setting time and increase of the film thickness, potentially jeopardizing their clinical performance [16]. Similarly, when hydraulic sealers are exposed to high temperatures, they experience a reduction in their physical properties such as setting time and flowability [15, 17]. According to the manufacturer, this new sealer developed to be heat-resistant, exhibits lower viscosity when heated and is more radiopaque than its predecessor [18]. Bioceramic sealer penetration into dentinal tubules is essential to create a mechanical anchorage between the sealer and the dentinal tubules and chemical hydroxyapatite formation [19, 20]. In addition to entombing any residual microorganisms [21].

There is a general lack of information available concerning the capacity of tubule penetration of TotalFill HiFlow bioceramic (BC) sealer when used together with Dual Rinse continuous chelation strategy and warm filling technique. Thus, the aim of this study was to inspect the penetration depth of the new modified calcium silicate–based sealer into dentinal tubules under confocal laser scanning microscopy (CLSM) when used with different irrigations and warm filling technique. The null hypothesis was that there would be no difference between using two different regimens – namely NaOCl solution combined with an etidronate powder (Dual Rinse ® HEDP) and the classic NaOCl and EDTA irrigating sequence – in terms of the depth of sealer penetration.

## Methods

### Sample size

A power analysis was designed to have adequate power for conducting a two-sided statistical test of the null hypothesis, which was that no difference would exist between the tested groups. By adopting an alpha (α) level of 0.05 and a beta (β) of 0.05 (i.e., power = 95%), an effect size (d) of 2.01 was calculated based on the results of a previous study [22]; the predicted sample size (n) was 16 (i.e., eight samples per group). To account for any sample loss, a sample size of n = 10 for each group was selected. The sample size calculation was performed using G*Power version 3.1.9.7.

### Sample selection

After the study protocol was approved by the ethical committee at the Faculty of Dentistry, Cairo University, Egypt (approval no. 211,022), Twenty single-rooted human mandibular premolars with a single root canal were collected from the university’s Department of Oral Surgery. Teeth were extracted for periodontal reasons. The teeth were cleaned of calculus and debris and examined under a surgical operating microscope (OMS 2350, Zumax Company, China) for caries, fractures, calcifications, cracks, and resorptions. Preoperative radiographs from both the buccolingual and mesiodistal directions were taken to ensure the presence of a single canal. The inclusion criteria were complete root formation, no calcification, and no internal or external root resorption. Teeth with a root curvature of 0°–10°, as measured using Schneider’s method, were selected for this study [23]. Teeth were excluded if they had external defects, incompletely formed apices or apices larger than a #20 K-type file, or previous root canal treatment.

The teeth were stored in normal saline solution containing 0.1% sodium azide. The crowns were sectioned with a 0.3-mm isomet saw (Isomet; Buhler Ltd, Lake Bluff, NY) with water cooling and the root canal length was standardised at 14 mm. Patency was verified by inserting a 10 K file (Dentsply Sirona, Tulsa, OK) into the canal space until the tip was visible at the apical foramen. The working length (WL) was calculated by subtracting 1 mm from this measurement.

### Root canal instrumentation

WaveOne Gold Glider reciprocating single files (15/ 0.02 Variable Taper; Dentsply Sirona, Tulsa, OK) were used to perform a mechanical glide path. Wave One Gold Medium (35/ 0.06) followed by WaveOne Gold Large (45/ 0.05; Dentsply Sirona, Tulsa, OK) were used for the mechanical instrumentation. All instruments were used in a slow in-and-out pecking motion mounted on an X-Smart plus endodontic motor (Dentsply Sirona, Tulsa, OK) set in the “WAVE ONE ALL” mode up to 1 mm from the WL.

The roots were randomly allocated into two equal groups (n = 10) using online random group allocation software (https://www.ramdomizer.org) according to the irrigation protocol. The groups were as follows:

Group I: NaOCl + EDTA.

Group II: NaOCl/Dual Rinse.

In group I, root canals were rinsed with 2 mL of 5.25% NaOCl at each instrument change using a plastic syringe with a 30-gauge side-vented needle (Max-i-Probe; Dentsply Rinn, Elgin, IL, USA) positioned 2 mm short of the working length. Once the mechanical preparation had been completed, final irrigation was performed with 5 mL of 5.25% NaOCl, followed by distilled water, and then 5 mL of 17% EDTA for 1 min and a final rinse with distilled water.

In group II (continuous chelation), the NaOCl/Dual Rinse ® HEDP solution was obtained by dissolving 0.9 g of Dual Rinse ® HEDP powder (mean content per capsule) in 10 mL of 5.25% NaOCl as per the manufacturer’s recommendations [24]. Mixtures were prepared immediately before treatment. Root canals were rinsed with 2 mL of NaOCl/Dual Rinse HEDP at each instrument change using a syringe with a 30-gauge side-vented needle (Max-i-Probe; Dentsply Rinn, Elgin, IL, USA), which was positioned 2 mm short of the WL. Once the mechanical preparation had been completed, final irrigation was performed using 10 mL of NaOCl/Dual Rinse HEDP followed by distilled water.

The canals were dried with absorbent points. To facilitate fluorescence under CLSM for measuring the penetration depth, TotalFill HiFlow BC sealer (FKG Dentaire) was mixed with fluorescent calcium indicator (Fluo-3; Thermo Fisher Scientific, USA). For standardisation, 1 g of endodontic sealer was weighed on an analytic scale (Adam Equipment Co. Ltd, MK10 0BD. UK) with an accuracy of 10 − 4 g. Next, 0.002 g of the Fluo-3 indicator was weighed at a ratio of 1:0.002 g (w/w) and mixed manually.

### Root canal obturation

WaveOne Gold conform fit gutta-percha points size large (45/05) master cone (Dentsply Maillefer, Switzerland) was used, and its fit was confirmed by taking a radiograph. The canal walls were coated with the sealer and obturated using a warm vertical compaction technique. The downpack procedure was performed with an EQ-V Pack (Meta Biomed Co. Ltd., Chungcheongbuk-do, Republic of Korea), which was set at 180 °C with a heater plugger size of 50/0.04 until reaching 3 mm from the WL, followed by compaction by hand pluggers (DiaDent, Cheongju, Republic of Korea)[17]. The backfill procedure was performed with the EQ-V fill handpiece of the EQ-V obturation unit (Meta Biomed Co. Ltd.) and 23-gauge needle tips containing gutta-percha at a temperature of 200 °C and condensed at the orifice level with hand pluggers.

Cavit (3 M ESPE; St Paul, MN, USA) was used to seal the access cavity; then, the teeth were stored for 4 weeks (37 °C, 100% relative humidity) to allow the sealer to completely set. To examine the sealer penetration depth, all roots were sectioned perpendicular to the long axis at depths of 3, 6, and 9 mm from the apex to represent the apical, middle, and coronal thirds, respectively, using a low-speed saw (Isomed, Buehler Ltd, Lake Bluff, IL, USA) under water cooling to a thickness of 1 ± 0.1 mm. The sections were polished with silicon carbide abrasive paper no. 500, 700, and 1200 (Arotec, Cotia, SP, Brazil) under water cooling to remove any residues from cutting.

### CLSM evaluation

**Fig. 1:**
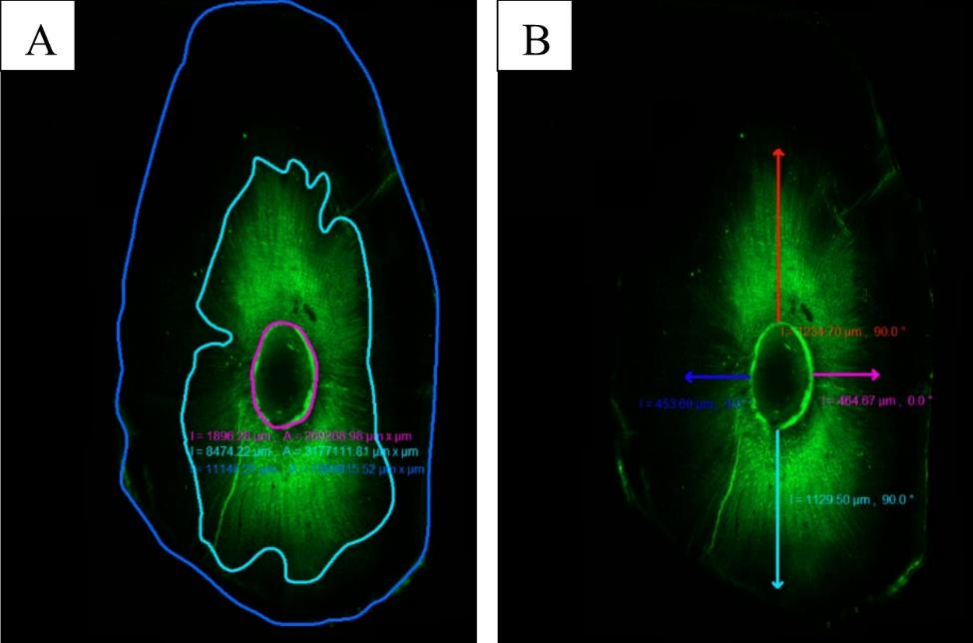
Representative confocal laser scanning microscopy images of (**a**) the whole area of sealer penetration, and (**b**) the maximum depth of penetration (µm) in the middle root section of the NaOCl/Dual Rinse HEDP group

Next, the samples were mounted on glass slides and examined under a confocal laser scanning microscope (CLSM 880, Carl Zeiss, GmbH, Jena, Germany) at 10× and the absorption and emission wavelengths of Fluo-3 indicator (559 nm). Using the ruler tool in the LSM image browser software package (Carl Zeiss Micro; Imaging GmbH, Jena, Germany), the percentage of the sealer penetration area was calculated by subtracting the amount of root canal space from the total area that the sealer penetrated; then, the values were converted into percentages. The maximum depth of penetration (µm) was measured from the canal wall to the point of maximum sealer penetration (Fig. 1). All root canals were prepared and obturated by the same experienced operator (N.R) to reduce inter-operator variability, Measurements were performed by one observer who was blinded to the groups (R.H) and repeated twice for interobserver reliability.

### Statistical analysis

Numerical data were presented as mean and standard deviation (SD) values. The Shapiro–Wilk test was used to test for normality, while the homogeneity of variances was tested using Levene’s test. The data revealed parametric distribution and variance homogeneity. They were analysed using a one-way ANOVA followed by Tukey’s post-hoc test for intergroup comparisons as well as repeated measures ANOVA followed by Bonferroni’s post-hoc test for intragroup comparisons. In all tests, the significance level was set at p < 0.05. Statistical analysis was performed with R version 4.1.3 for Windows [25].

## Results

**Fig. 2:**
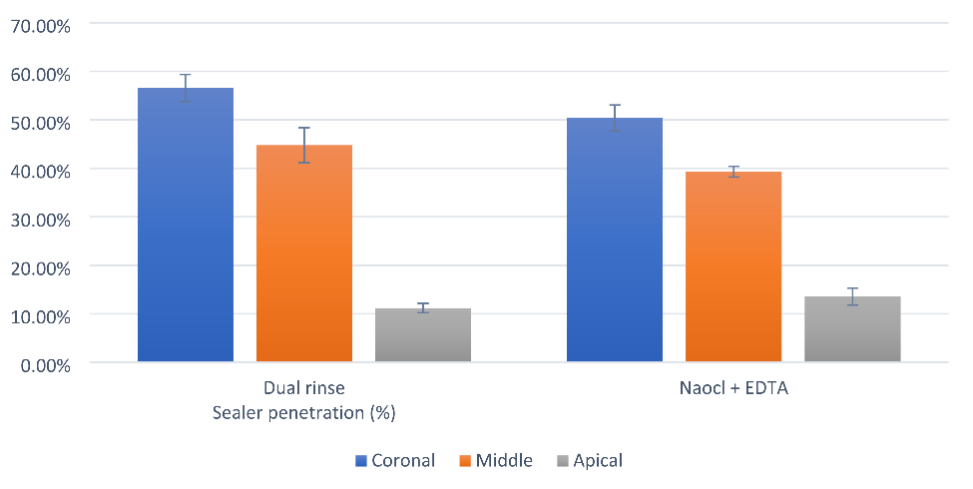
Bar chart presenting mean and standard deviation values for sealer penetration (%) in different root sections

For both groups, the results of the percentage of sealer penetration and the maximum depth of penetration exhibited significant differences between the values measured in different sections (p < 0.001; Table 1; Fig. 2). The coronal section had the highest value followed by the middle section, while the lowest value was measured at the apical section. Post-hoc pairwise comparisons revealed values measured at different sections to be significantly different from each other (p < 0.001).

Comparing the overall results of all the sections tested, no statistically significant difference existed between the groups regarding the percentage of sealer penetration (p = 0.612) and the maximum depth of penetration (p > 0.05; Table 1; Fig. 2). However, for the coronal section, the Dual Rinse group had a significantly higher percentage of sealer penetration (p < 0.05) and a greater depth of penetration than the NaOCl + EDTA group (p = 0.022). By contrast, for the apical section, the NaOCl + EDTA group had a significantly higher percentage of sealer penetration (p = 0.015) but no significant difference in the depth of penetration (p > 0.05).

**Table 1:** Inter- and intragroup comparisons for maximum depth of penetration (µm)

Section	Maximum depth (µm; mean ± SD)		p value
	Dual rinse	NaOCL + EDTA	
**Coronal**	700.49 ± 1.15^ A^	688.51 ± 10.81^ A^	0.022*
**Middle**	556.31 ± 25.81^B^	575.10 ± 14.57^B^	**0.151**
**Apical**	359.26 ± 25.96^ C^	359.25 ± 31.46^ C^	**1**
**p value**	< 0.001*	< 0.001*	
**Total**	538.69 ± 145.28	540.95 ± 141.91	**0.962**

Means with different superscript letters within the same vertical column are significantly different *significant (p < 0.05)

## Discussion

The penetration of root canal sealer into the dentinal tubules is a desired property as it can result in mechanical interlocking between the sealer and root dentin [26]. Many factors can influence the depth of penetration, including the structure of the dentin, the irrigation solution used, the efficiency of smear layer removal, and the varied physical and chemical properties of the sealer [27, 28]. The warm obturation techniques with bioceramic sealers have drawn some criticism. The use of heat during the thermoplasticized obturation techniques resulted in significant alterations in the properties of the bioceramic sealers [29]. The temperature increase may also affect the biomineralization process and the Apatite-forming capacity [16]. Therefore, this in vitro study aimed to assess the dentinal tubule penetration of TotalFill HiFlow BC sealer when used together with Dual Rinse continuous chelation strategy and warm filling technique using CLSM.

CLSM analysis was used to assess sealer penetration through the dentinal tubules. CLSM studies often measure the maximum sealer penetration depth and the percentage of sealer penetration in relation to the entire root diameter [30]. CLSM offers multiple advantages compared with other magnification methods; for example, it can display the situation at different levels under the specimen’s surface rather than evaluate the surface of the specimen. Thus, CSLM does not require any surface preparation that could cause artifacts and is not dependent on surface quality [31].

Fluo-3 indicator was used as a fluorophore to assess the extent of penetration of the calcium silicate–based sealer. It was previously reported that the use of rhodamine B with calcium silicate–based sealers leads to false-positive results in terms of the interpretation of penetration data. Rhodamine B binds with the water necessary for chemical reactions during the setting of the sealer and tubular humidity, as opposed to binding with the bioceramic sealer. Thus, fluorescent-tagged regions would not necessarily be filled with bioceramic sealer but with water inside the dentinal tubules, which is responsible for carrying the fluorophore, impairing the validity of earlier investigations [32]. By contrast, Fluo-3, a nonfluorescent compound, becomes substantially more fluorescent once it binds to the calcium in calcium silicate–based sealers, which makes it easier to assess the sealer’s penetration with CLSM [33].

Sealer penetration was significantly higher in the coronal third of the root canals than it was in the middle and apical thirds, as well as significantly higher in the middle than the apical thirds (p < 0.001). This is in agreement with a previous study [34]. The presence of dentinal tubules at higher densities in the coronal and middle thirds versus the apical third could be responsible for the decreasing penetration values from the coronal to the apical regions [35]. The ineffective delivery of irrigant to the apical third of the canal, the smaller diameter, and the reduction in the number of dentinal tubules in this region may explain the poorer sealer penetration in the apical third [27]. Areas of sclerotic dentin are more dominant, rendering the apical region of the tooth less permeable than the coronal region [36, 37]. According to Alegre et al. [38], the differing pressures, depth, and heat of the pluggers applied during obturation could also explain the lower degree of sealer penetration in the apical third rather than the coronal third.

By applying the continuous chelation concept through irrigation with NaOCl/Dual Rinse HEDP during chemo-mechanical preparation, this resulted in the highest sealer penetration values in the coronal and middle thirds. This result is in line with the findings of Ulusoy et al. [39], who found that HEBP had a higher smear layer removal capacity than EDTA. This could be rationalised by the fact that the continuous chelation reduces the accumulation of hard tissue debris during root canal instrumentation, which eventually prevents or decreases smear layer formation [9]. Additionally, HEBP does not affect the hydration properties of calcium silicate cement [12]. EDTA, on the other hand, inhibits the hydration of tricalcium silicate cement by chelating calcium ions released from the tricalcium complex, which is the principal ingredient of MTA [40]. This could also explain the higher sealer penetration observed in the NaOCl/EDTA group in the apical third due to the strong chelating effect of EDTA, which can produce a demineralised dentin zone that is too deep compared with Dual Rinse HEDP [41].

Gawdat and Bedier [22] found that group (NaOCl/Dual Rinse) displayed better sealer penetration inside the dentinal tubules than group (NaOCl/EDTA), this contradiction in the results with the current study could be contributed to the differences in the methodology, where they used Bioceramic-based sealer (Well-Root ST sealer; Vericom, Gangwon-Do, Korea) mixed with rhodamine B dye (Sigma-Aldrich, St. Louis, MO, USA), in a single-cone obturation technique.

A higher percentage of sealer penetration denotes a higher percentage of sealed tubules, which could lead to greater entrapment of microorganisms, increasing the chances of successful treatment. Additionally, a deeper level of penetration may cause the sealer to encounter more microorganisms in each tubule, thus extending the antiseptic effect of the sealer especially in the apical third where the ineffective delivery of irrigant to the apical third of the canal occur [33].

The current investigation is limited by the fact that it was a pure laboratory study. However, clinical situations were simulated. While authors of this study tried to eliminate technical variables, however, one of the limitations of this study was that the extracted tooth model does not provide a uniform cross-section of all samples which might have affected the adaption of the root canal filling to canal walls and sealer penetration circumferentially. Although randomization of the samples was done to minimize selection bias and the impact of heterogeneity of cross-sections. Further research employing different sonic and ultrasonic activation is necessary, possibly allowing for deeper disinfection and sealer penetration are required.

## Conclusions

With both types of irrigation used, dentinal tubule penetration was higher in the coronal section than in the apical section. Continuous chelation using NaOCl/Dual Rinse HEDP performed better in the coronal segments, while irrigation using NaOCl + EDTA promoted a higher percentage of sealer penetration in the apical segment.

## Data Availability

The datasets used and/or analyzed during the current study are available from the corresponding author on reasonable request.
